# Intercropping with *Paris polyphylla* and *Ganoderma lucidum*: regulatory effects on the rhizosphere microbial community and the quality of *Polygonatum cyrtonema*

**DOI:** 10.3389/fmicb.2025.1711104

**Published:** 2025-12-17

**Authors:** Huiyong Zheng, Yanming Zhu, Penghui Liao, Fengfang Lin, Wei Ye, Fanghua Mao, Liang Fang, Yanghui Fang, Hailan Su

**Affiliations:** 1 Institute of Crop Sciences, Fujian Academy of Agricultural Sciences (Fujian Germplasm Resources Center), Fuzhou, China; 2 Digital Agriculture Institute, Fujian Academy of Agricultural Sciences, Fuzhou, China; 3 Fujian Forestry Science and Technology Extension General Station, Fuzhou, China; 4 Sanming Academy of Agricultural Sciences, Sanming, China; 5 College of Forestry, Fujian Agriculture and Forestry University, Fuzhou, China

**Keywords:** *Polygonatum cyrtonema*, forest-based cultivation, rhizosphere microbial communities, nutrient-microbe interactions, polysaccharides

## Abstract

**Introduction:**

Optimizing the cultivation of *Polygonatum cyrtonema* requires a comprehensive understanding of how intercropping influences both its quality and the rhizosphere microenvironment.

**Methods:**

This study conducted a comparative analysis between monoculture cultivation of *P. cyrtonema* (PC) and two intercropping systems: *P. cyrtonema* with *Paris polyphylla* (PCPP) and with *Ganoderma lucidum* (PCG). The investigation assessed the influence of these cultivation systems on yield, quality attributes, soil physicochemical characteristics, and rhizosphere microbial communities.

**Results:**

Both intercropping systems significantly enhanced nutrient accumulation in the rhizosphere. Specifically, the PCG system increased available potassium by 72.76%, while the PCPP system elevated alkali-hydrolyzable nitrogen and total nitrogen by 7.19% and 7.02%, respectively, relative to PC. Additionally, both intercropping systems increased the relative abundance of microbial taxa such as *Furcasterigmium* and *unclassified_f__Mortierellaceae*. The PCPP system additionally promoted the proliferation of beneficial microorganisms, including *Burkholderia-Caballeronia-Paraburkholderia, Rhodomicrobium*, and *norank_c_AD3*. Available potassium and phosphorus were identified as key factors driving alterations in the rhizosphere microbial community structure. Although intercropping did not significantly affect *P. cyrtonema* yield, the PCPP system improved water-soluble extract content by 2.8% and polysaccharide content by 12.1% compared to monoculture.

**Discussion:**

These findings indicate that intercropping modulates *P. cyrtonema* quality through synergistic interactions between soil nutrients and microbial community composition, with the PCPP system recommended as an optimal approach for integrated forest-based cultivation.

## 1 Introduction

*Polygonatum cyrtonema*, commonly known as giant Chinese Solomon's seal, is valued for its medicinal and culinary uses due to its rhizomes, which contain bioactive compounds such as polysaccharides, steroidal saponins, and flavonoids. These compounds provide immunomodulatory, antioxidant, anti-fatigue, and glycemic regulatory effects. Despite its widespread use in the pharmaceutical and functional food industries, continuous monoculture cultivation has led to soil nutrient depletion, altered microbial community structures, and the accumulation of soil-borne pathogens. These factors collectively impair plant health and reduce both yield and quality. Therefore, sustainable cultivation strategies that preserve soil health and crop productivity are essential.

Intercropping is widely recognized as an environmentally sustainable agricultural practice that enhances resource use efficiency, improves soil fertility, and increases crop yields (Schütz et al., 2022). Empirical evidence indicates that intercropping diversifies nutrient sources for soil microorganisms and promotes a more robust and beneficial microbial community structure (Ilakiya et al., 2023), which can positively influence the accumulation of bioactive compounds in medicinal plants (Zhou et al., 2023; Duan et al., 2024; Han et al., 2025).

However, prior research on *P. cyrtonema* intercropping has predominantly focused on yield and soil nutrients changes (Xu et al., 2023; Ke et al., 2018), with limited insights into how intercropping affects rhizosphere microbial community structure and function, and the key soil nutrients driving these changes. Moreover, the mechanistic links between soil microbial alterations and bioactive compound accumulation in *P. cyrtonema* rhizomes remain unclear (Zhou et al., 2023; Jiang et al., 2024).

This study aims to elucidate the relationships among intercropping patterns, soil nutrients, microbial communities, and rhizome quality, by examining *P. cyrtonema* growth under monoculture and intercropping with two companion species: *Paris polyphylla* and *Ganoderma lucidum*. *P. polyphylla*, noted for shade tolerance, releases amino acid-rich root exudates that promote soil nitrogen cycling (Zheng et al., 2020), while *G. lucidum*, a medicinal fungus, secretes organic acids enhancing soil fertility (Zou et al., 2022; Han et al., 2022; Wen et al., 2023). The complementary ecological functions of these species offer a promising approach to optimizing the rhizosphere environment of *P. cyrtonema*.

**Figure 1:**
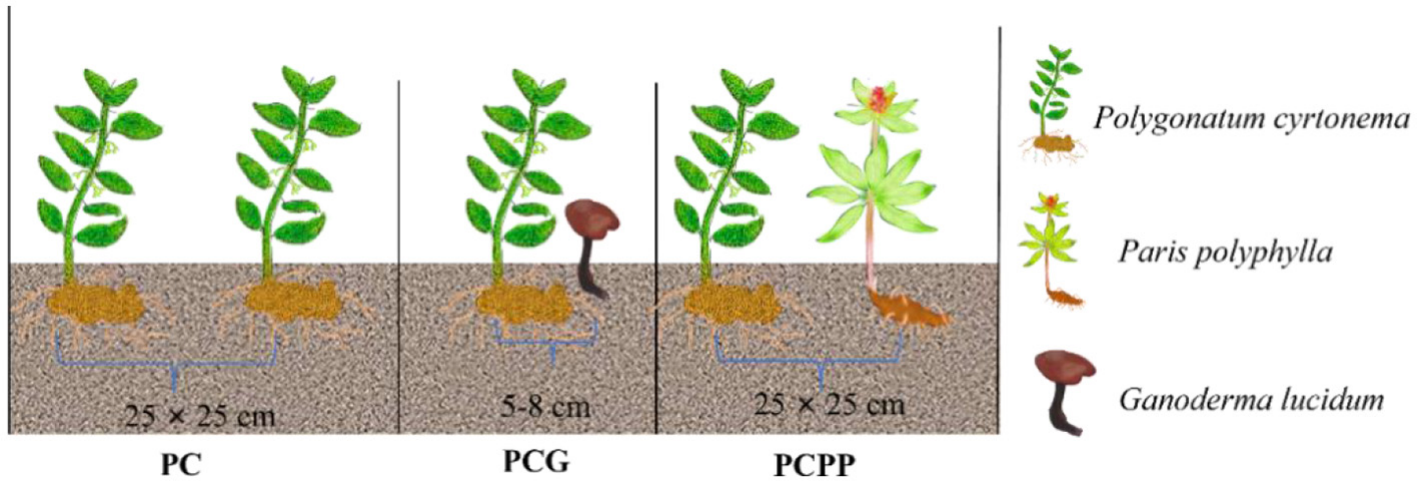
The different intercropping treatments and planting configurations used in this study. The monoculture system of *P. cyrtonema* (PC) was compared with two intercropping treatments: *P. cyrtonema* with *P. polyphylla* (PCPP) and *P. cyrtonema* with *G. lucidum* (PCG). Each treatment featured distinct plant spacing and root configurations, with the spacing between plants shown as 25 × 25 cm for PC and PCPP systems, while 5-8 cm for PCG system.

We hypothesized that intercropping *P. cyrtonema* with *P. polyphylla* or *G. lucidum* modifies rhizosphere nutrient availability, thereby improving microbial community structure and promoting bioactive compound accumulation in rhizomes. To test this, three cultivation systems were established: monoculture of *P. cyrtonema* (PC), intercropping with *P. polyphylla* (PCPP), and intercropping with *G. lucidum* (PCG) (Figure 1). The study objectives were to (1) assess the effects of intercropping on soil nutrient parameters including available potassium (AK), alkali-hydrolyzable nitrogen (AHN), and available phosphorus (AP); (2) characterize changes in rhizosphere microbial community structure and functional groups; and (3) explore interrelationships among soil nutrients, microbial communities, and *P. cyrtonema* rhizome quality. The findings aim to advance understanding of plant-soil-microbe interactions critical for effective intercropping and provide a scientific basis for sustainable high-quality cultivation of *P. cyrtonema*.

## 2 Materials and methods

### 2.1 Site description and experimental design

The field experiment was conducted at the Taozhou experimental platform in Guangze County, Nanping City, Fujian Province, China (27°18′N, 117°E; elevation 298 m). The site is characterized by an annual accumulated temperature of 6336 °C and average annual precipitation of 1864 mm, with sandy loam soil predominating (Su et al., 2024). Three year old seedlings of *P. cyrtonema* and *P. polyphylla* were used, cultivated continuously for 2 years prior to harvest. *G. lucidum* was cultivated using fungus bags producing fruiting bodies annually for 2 years. Soil preparation included ridging and uniform application of decomposed organic fertilizer (30,000 kg·hm^−2^) and superphosphate (750 kg·hm^−2^). Treatments were arranged in plots (1.0 m width, 0.30 m height) with 0.3 m spacing and drainage trenches. In March 2022, three planting systems were established: monoculture of *P. cyrtonema* (PC), intercropping of *P. cyrtonema* with *P. polyphylla* (PCPP), and intercropping of *P. cyrtonema* with *G. lucidum* (PCG) (Figure 1). A randomized complete block design (RCBD) was adopted with 3 replicates, resulting in a total of 9 plots. A 1 meter isolation belt was set between adjacent plots, and 1-meter guard rows were arranged around the entire experimental area to minimize edge effects and prevent cross-contamination of fertilizers and irrigation water. Each plot consisted of 100 plants. Plant spacing was 25 × 25 cm for PC and PCPP, and 5–8 cm between *P. cyrtonema* and *G. lucidum* cultivation sticks in PCG. Weeding occurred thrice annually with concurrent organic fertilizer application (30,000 kg·hm^−2^). All treatments received identical field management.

### 2.2 Soil and plant sampling

In October 2024, after 2 years of cultivation, ten vigorous *P. cyrtonema* plants per plot were randomly selected. After shaking off the bulk soil (non-rhizosphere soil) adhering to the rhizomes, the soil attached to the rhizome surface (with a thickness of approximately 1–2 mm) was gently brushed off using a sterile brush, which was defined as rhizosphere soil. Impurities were removed from the 10 rhizosphere soil subsamples obtained from each plot: every 3 subsamples were combined into one composite sample, and the remaining 4 subsamples were merged into another composite sample, resulting in a total of 3 composite rhizosphere soil samples per plot. Therefore, a total of 9 soil samples were obtained from the 3 plots of each treatment. Then, each composite rhizosphere soil sample was evenly divided into two portions: one portion was rapidly frozen in liquid nitrogen for subsequent molecular analysis. Soil samples were flash-frozen in liquid nitrogen and stored at −80 °C for microbial analysis. The remaining samples were air-dried, ground, and sieved for physicochemical analyses. Agronomic traits were measured *in situ*, belowground plant were washed, dried at 60 °C, ground, and sieved through a 60-mesh screen for further analyses.

### 2.3 Measurement of agronomic traits

Morphological parameters including plant height, stem diameter, leaf length and width, and leaf number were measured post cessation of aboveground growth (August 2024). In October 2024, fresh weights of aerial parts, rhizome biomass, rhizome dimensions, and white root count and proportion of white roots (i.e., white new roots, calculated as the number of white roots divided by the total root count) were recorded. The proportion of white roots serves as an indicator of root activity, nutrient absorption, and overall plant vigor, reflecting root adaptability and soil environment impacts under different intercropping systems (Baldi et al., 2010).

### 2.4 Determination of ethanol-soluble extractives and polysaccharides in *P. cyrtonema*

The content of ethanol-soluble extractives was quantified via hot extraction per the Chinese Pharmacopeia (State Pharmacopeia Commission of the People's Republic of China, 2020). The content of ethanol-soluble extractives (%) = (Total weight of the tested extractives/Mass of the stems used for extraction) × 100%. Polysaccharide content was measured using the phenol-sulfuric acid assay following SN/T 4260-2015 standards (SN/T 4260, 2015).

### 2.5 Soil physicochemical analyses

Soil pH was measured with a pH meter. Soil organic matter (SOM) was determined by the potassium dichromate-sulfuric acid oxidation method as described by Chen F. et al. (2023). Alkali-hydrolyzable nitrogen (AHN) and total nitrogen (TN) were quantified via Kjeldahl and automatic nitrogen analyzer methods, respectively (Chen J. et al., 2023). Available phosphorus (AP) was extracted with ammonium fluoride-hydrochloric acid and determined colorimetrically (Chen F. et al., 2023). Available potassium (AK) was assessed by ammonium acetate extraction and flame photometry (Chen F. et al., 2023). Total phosphorus (TP) and total potassium (TK) were analyzed after acid digestion with nitric acid and perchloric acid, followed by hydrofluoric acid decomposition and measurement via flame photometry, according to Bao (2000).

### 2.6 Soil microbial DNA extraction and amplicon sequencing

Genomic DNA was extracted from soil samples using the E.Z.N.A.^®^ Soil DNA Kit (Omega Bio-tek, Norcross, GA, USA). Sequencing was performed by Shanghai Majorbio Biopharm Technology Co., Ltd. DNA quality and concentration were assessed by 1% agarose gel electrophoresis and NanoDrop 2000 spectrophotometer (Thermo Fisher Scientific, USA). The fungal internal transcribed spacer (ITS) region was amplified using ITS1F (5′-CTTGGTCATTTAGAGGAAGTAA-3′) and ITS2R (5′-GCTGCGTTCTTCATCGATGC-3′) primers, while the bacterial 16S rRNA gene V3–V4 region was amplified with primers 338F (5′-ACTCCTACGGGAGGCAGCAG3′) and 806R (5′-GGACTACHVGGGTWTCTAAT3′). PCR products were purified using a PCR clean-up kit (YuHua, Shanghai, China) and quantified with a Qubit 4 Fluorometer (Thermo Fisher Scientific, USA). Library preparation followed NEXTFLEX Rapid DNA-Seq Kit protocol (Li et al., 2025), and sequencing was conducted on the Illumina PE300 platform. The raw sequencing data have been deposited in the NCBI Sequence Read Archive (SRA) database.

### 2.7 Data analysis

Prior to conducting analysis of variance (ANOVA) on the agronomic traits of plants, soil physicochemical properties, and rhizome constituents among different treatments, the Shapiro-Wilk test was employed to verify the normality of the data (>0.05 indicates that the data conform to a normal distribution), and only data that met the normality assumption were subjected to subsequent ANOVA (Chang et al., 2022). Statistical differences in agronomic traits, soil physicochemical properties, and rhizome constituents among treatments were evaluated using the Least Significant Difference (LSD) test implemented in DPS 18.10 software (Gu et al., 2023). Microbial diversity analyses were performed on the Majorbio Cloud Platform (https://cloud.majorbio.com; Gu et al., 2023). Alpha diversity indices, reflecting community richness and evenness, were calculated using Mothur version 1.30.2 (Schloss et al., 2009). Beta diversity was assessed via Analysis of Similarities (ANOSIM) to compare microbial community structures across treatments, with significant taxonomic differences identified through intergroup comparisons. Pearson correlation analysis was conducted to explore relationships among soil physicochemical parameters, rhizome biomass, fresh rhizome weight, active ingredient concentrations, and microbial phyla. Sankey diagrams were constructed using Origin 2024, and microbial co-occurrence networks were established based on Spearman's correlation coefficients among microbial taxa (Duan et al., 2024). Redundancy analysis (RDA) was employed to evaluate the influence of soil properties on microbial community composition at the phylum level, following the approach of Zou et al. (2022).

## 3 Results

### 3.1 Agronomic performance of *P. cyrtonema*

**Figure 2:**
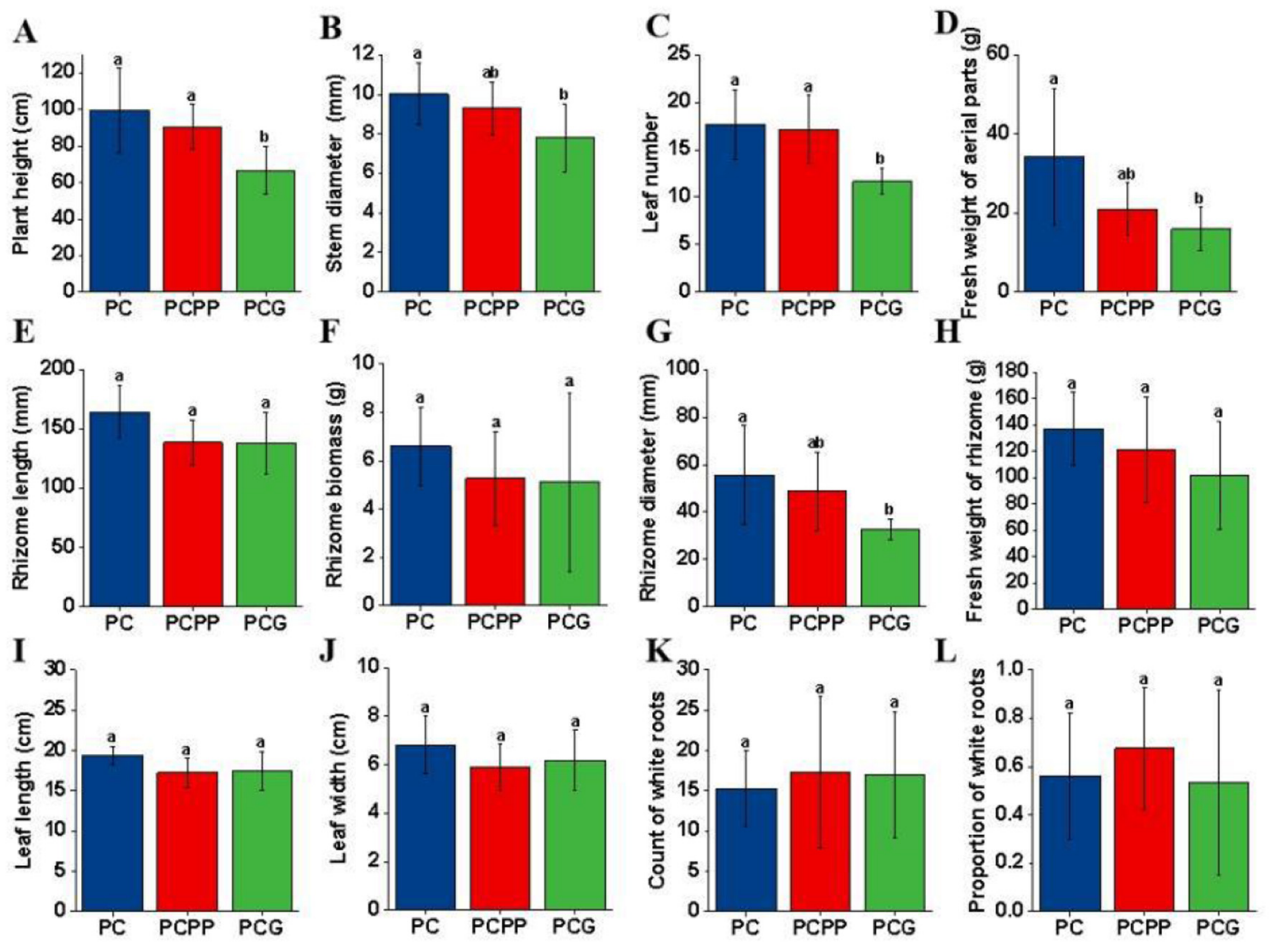
Agronomic traits of *P. cyrtonema* under different intercropping systems. **(A)** Plant height; **(B)** Stem diameter; **(C)** Leaf number; **(D)** Fresh weight of aerial parts; **(E)** Rhizome length; **(F)** Rhizome biomass; **(G)** Rhizome diameter; **(H)** Fresh weight of rhizome; **(I)** Leaf length; **(J)** Leaf width; **(K)** Count of white roots; **(L)** Proportion of white roots. PC, Monoculture of *P. cyrtonema*; PCPP, Intercropping of *P. cyrtonema* and *P. polyphylla*; PCG, Intercropping of *P. cyrtonema* and *G. lucidum*. Different lowercase letters within the same column indicate statistically significant differences (*P* < 0.05).

Significant differences in *P. cyrtonema* agronomic traits were observed among cultivation systems (Figure 2). The PC system exhibited greater plant height, stem diameter, leaf number, fresh weight of aerial parts, and rhizome diameter compared to PCG (*P* < 0.05, Figures 2A–D, G), whereas no significant differences were found between PC and PCPP. Rhizome length, rhizome biomass and fresh weight, leaf length and width, number of white roots, and the proportion of white roots did not differ significantly among treatments (Figures 2E, F, H–L).

### 3.2 Active compound contents in *P. cyrtonema*

The PCPP system yielded the highest ethanol-soluble extractives (80.30%) and polysaccharide content (9.73 g·100g^−2^, dry weight), surpassing PC by 2.82% and 12.10%, respectively. The PCG system exhibited the lowest levels, with ethanol-soluble extractives and polysaccharides measuring 75.60% and 8.40 g·100g^−2^, respectively. These results suggest that the PCPP cultivation system may enhance the accumulation of ethanol-soluble extractives and polysaccharides in *P. cyrtonema* (Table 1).

**Table 1:** Contents of active ingredients of *P. cyrtonema* under different intercropping systems.

**Samples**	**Ethanol-soluble extractives (%)**	**Polysaccharides(g·100g^−1^ DW) ^c^**
PC^a^	78.10 ± 0.10b^b^	8.68 ± 0.30b
PCPP	80.30 ± 0.10a	9.73 ± 0.20a
PCG	75.60 ± 0.01c	8.40 ± 0.20b

^a^ PC: Monoculture of *P. cyrtonema*; PCPP: Intercropping of *P. cyrtonema* and *P. polyphylla*; PCG: Intercropping of *P. cyrtonema* and *G. lucidum*. ^b^ Different lowercase letters within the same column indicate statistically significant differences (*P* < 0.05). ^c^ DW stands for dry weight.

### 3.3 Physicochemical properties of rhizosphere soil

**Figure 3:**
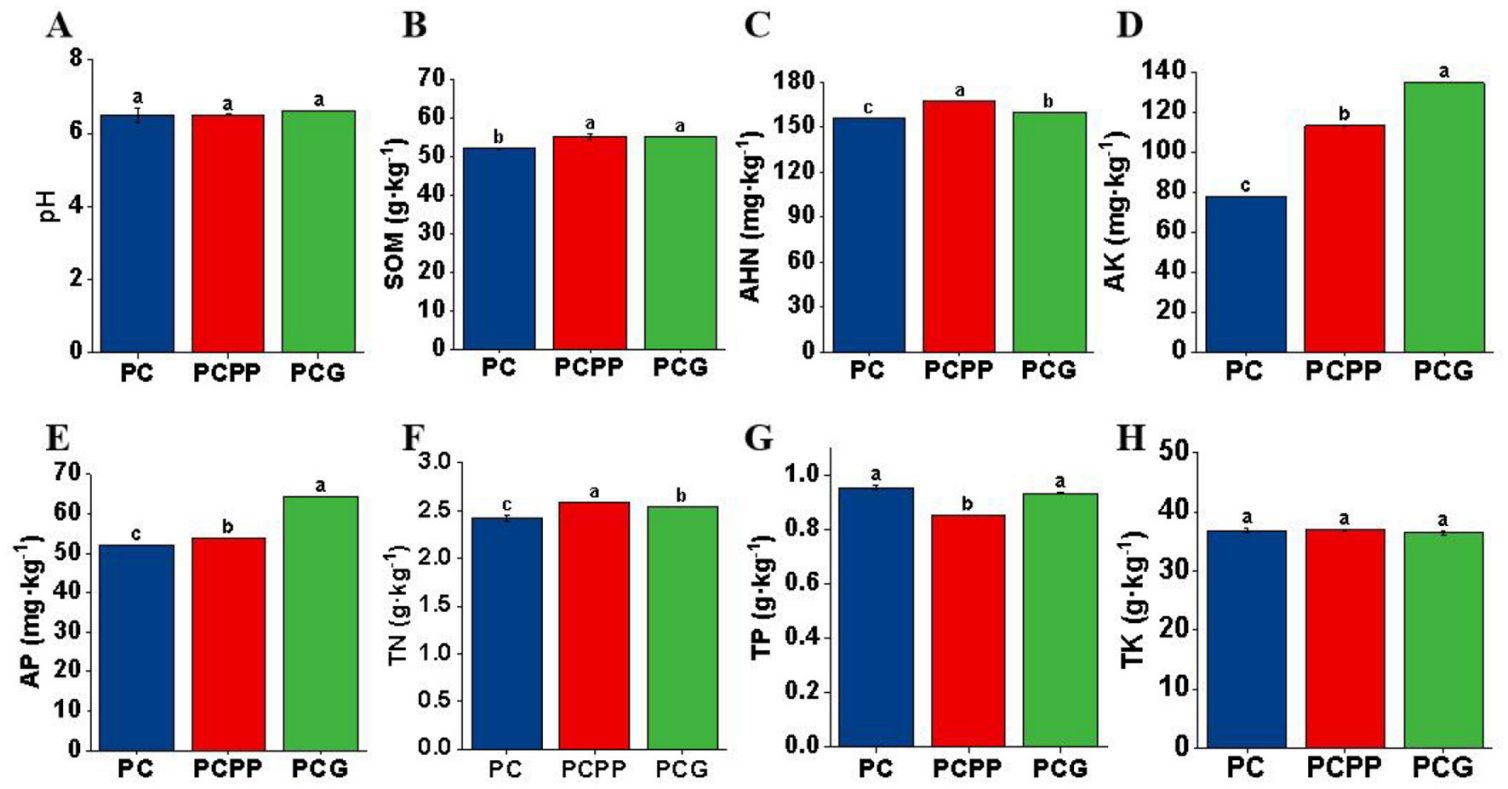
Physicochemical properties of rhizosphere soil of *P. cyrtonema* cultivated under various intercropping systems. **(A)** Soil pH (pH); **(B)** Soil organic matter (SOM); **(C)** Alkali-hydrolyzable nitrogen (AHN); **(D)** Available potassium (AK); **(E)** Available phosphorus (AP); **(F)** Total nitrogen (TN); **(G)** Total phosphorus (TP); **(H)** Total potassium (TK). PC, Monoculture of *P. cyrtonema*; PCPP, Intercropping of *P. cyrtonema* and *P. polyphylla*; PCG, Intercropping of *P. cyrtonema* and *G. lucidum*. Different lowercase letters indicate significant differences (*P* < 0.05).

The pH values of rhizosphere soil across all treatment groups ranged from 6.49 to 6.60, with no statistically significant differences detected among the systems (Figure 3A). Compared to the PC system, both the PCPP and PCG treatments led to significant increases in soil organic matter (SOM), alkali-hydrolyzable nitrogen (AHN), available potassium (AK), available phosphorus (AP), and total nitrogen (TN). Specifically, the PCPP treatment enhanced SOM, AHN, AP, AK, and TN by 6.07%, 7.19%, 2.89%, 45.09%, and 7.02%, respectively, whereas the PCG treatment resulted in increases of 5.94%, 2.54%, 23.20%, 72.76%, and 4.96% in these parameters (Figures 3B–F). Notably, AHN and TN concentrations were significantly higher under the PCPP compared to PCG (Figures 3C, F), while AP and AK levels were significantly elevated in PCG relative to PCPP (Figures 3D, E). No significant differences in SOM content were observed between the PCPP and PCG treatments (Figure 3B). Total phosphorus (TP) content was significantly lower in PCPP compared to both PC and PCG, with no significant variation between PC and PCG (Figure 3G). Total potassium (TK) levels did not differ significantly among the treatments (Figure 3H). Collectively, both PCPP and PCG treatments promoted the accumulation of SOM, AHN, AP, AK, and TN relative to monoculture conditions. The PCPP treatment exhibited a distinct propensity to enhance AHN and TN accumulation, whereas the PCG treatment preferentially facilitated the enrichment of AP and AK.

### 3.4 Diversity of rhizosphere soil microbial communities

A total of 554,738 high-quality fungal sequences and 529,562 high-quality bacterial sequences were obtained from nine soil samples to assess the diversity of soil microbial communities. Analysis of fungal alpha diversity indices, including Ace, Chao, Shannon, Simpson, and Sobs, revealed no statistically significant differences among the PC, PCPP, and PCG systems (Supplementary Table S1). Nonetheless, there was a discernible trend toward increased richness, with the Ace and Chao indices ranking in the order PCG > PCPP > PC. Beta diversity analysis indicated that the fungal community structures of PCPP and PCG were similar to each other but distinct from that of PC (Supplementary Figure S1A).

Regarding bacterial communities, alpha diversity indices (Ace, Chao, Shannon, Simpson, and Sobs) similarly showed no significant differences across the three cultivation systems (Supplementary Table S1). However, the indices Ace, Chao, Shannon, and Sobs followed the ranking PC > PCG > PCPP. Beta diversity analysis revealed significant differences in bacterial community composition between PC and PCPP, as well as between PC and PCG (Supplementary Figure S1B).

### 3.5 Composition of the rhizosphere soil microbial community

**Figure 4:**
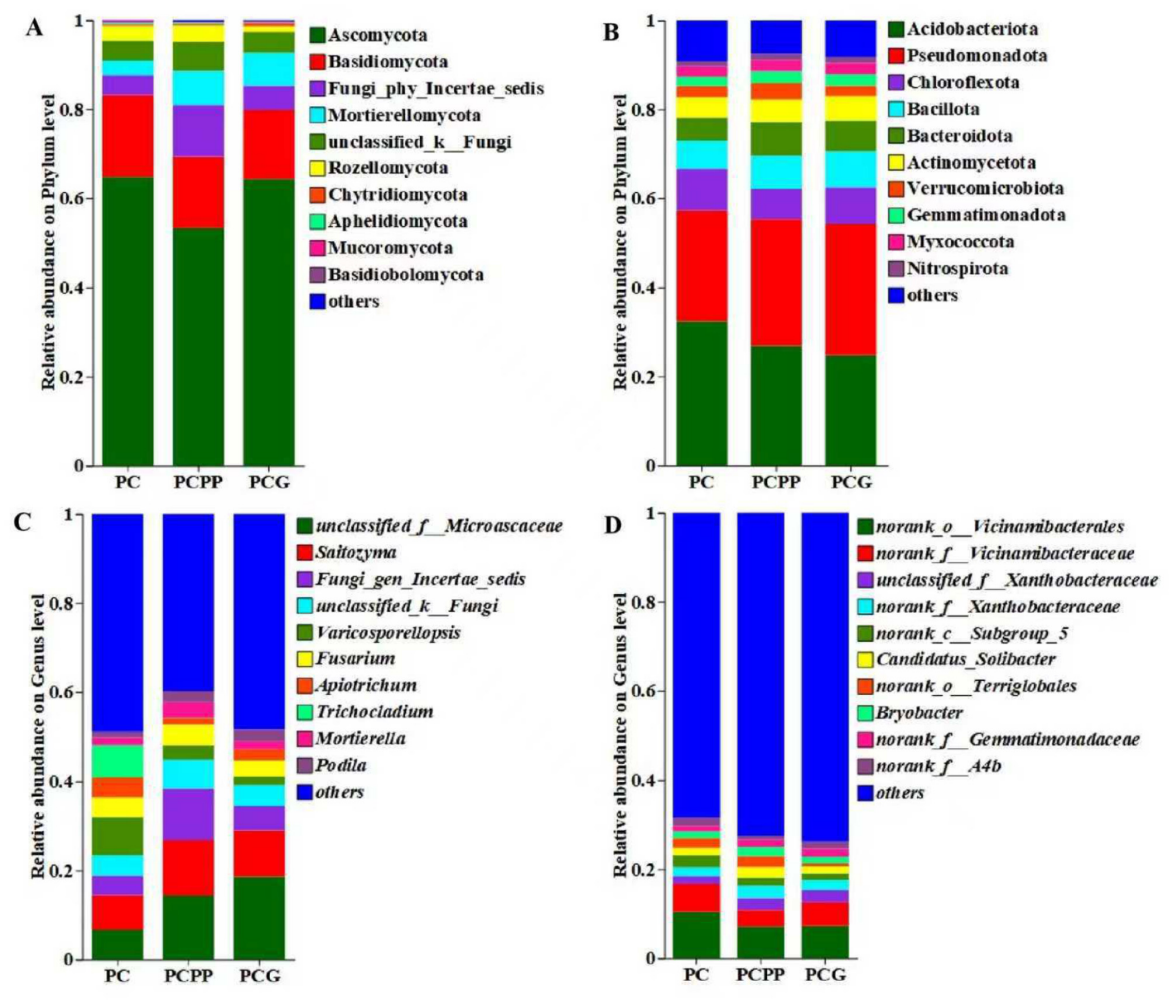
The effects of different intercropping patterns on the composition of fungal species in the rhizosphere soil of *P. cyrtonema*. **(A)** Fungal phylum level; **(B)** Bacterial phylum level; **(C)** Fungal genus level; **(D)** Bacterial genus level. PC, Monoculture of *P. cyrtonema*; PCPP, Intercropping of *P. cyrtonema* and *P. polyphylla*; PCG, Intercropping of *P. cyrtonema* and *G. lucidum*.

The composition of the soil microbial community was comprehensively characterized, identifying 3,907 fungal operational taxonomic units (OTUs) spanning 17 phyla and 824 genera. The analysis concentrated on the ten most abundant fungal phyla across the three distinct cultivation systems. In the monoculture system (PC), Ascomycota, (64.81%) and Basidiomycota (18.39%) were the predominant phyla (Figure 4A). Similar patterns were observed in the PCPP and PCG systems, where Ascomycota and Basidiomycota remained dominant. However, the relative abundances of other phyla, such as Fungi_phy_Incertae_sedis, Mortierellomycota, and Rozellomycota, varied among the cultivation systems (Figure 4A). At the genus level, the ten most prevalent fungal genera included *unclassified_f__Microascaceae, Saitozyma, Fungi_gen_Incertae_sedi*s, *unclassified_k__Fungi, Varicosporellopsis, Fusarium, Apiotrichum, Trichocladium, Mortierella*, and *Podila* (Figure 4C). Within the PC system, eight genera exhibited relative abundances exceeding 2%, notably *Varicosporellopsis* (8.61%), *Saitozyma* (7.72%), and *Trichocladium* (7.20%) (Figure 4C). Similarly, in the PCPP and PCG systems, several genera surpassed the 2% relative abundance threshold, including *unclassified_f__Microascaceae, Saitozyma*, and *Fungi_gen_Incertae_sedis*, with their abundances differing across the cultivation systems (Figure 4C).

Concerning bacterial communities, a total of 9,298 bacterial OTUs were identified, distributed among 47 phyla and 1,042 genera. The dominant bacterial phyla comprised Acidobacteriota (24.48% to 32.42%), Pseudomonadota (24.91% to 29.48%), Chloroflexota (6.73% to 9.34%), Bacillota (6.29% to 8.07%), Bacteroidota (5.18% to 7.59%), Actinomycetota (4.56% to 5.57%), Verrucomicrobiota (2.31% to 3.59%), Gemmatimonadota (2.08% to 2.68%), Myxococcota (2.27% to 2.50%), and Nitrospirota (1.10% to 1.50%) (Figure 4B). At the genus level, prominent bacterial taxa included *norank_o_Vicinamibacterales* (7.12% to 10.49%), *norank_f_Vicinamibacteraceae* (3.72% to 6.12%), *unclassified_f_Xanthobacteraceae* (1.81% to 2.70%), *norank_f_Xanthobacteraceae* (1.98% to 2.76%), *norank_c_Subgroup_5* (1.45% to 2.69%), *Candidatus_Solibacter* (1.54% to 2.39%), *norank_o_Terriglobales* (1.74% to 2.52%), *Bryobacter* (1.47% to 1.93%), *norank_f_Gemmatimonadaceae* (1.28% to 1.80%), and *norank_f_A4b* (1.84% to 1.88%) (Figure 4D).

### 3.6 Variations in microbial community composition of rhizosphere soil

**Figure 5:**
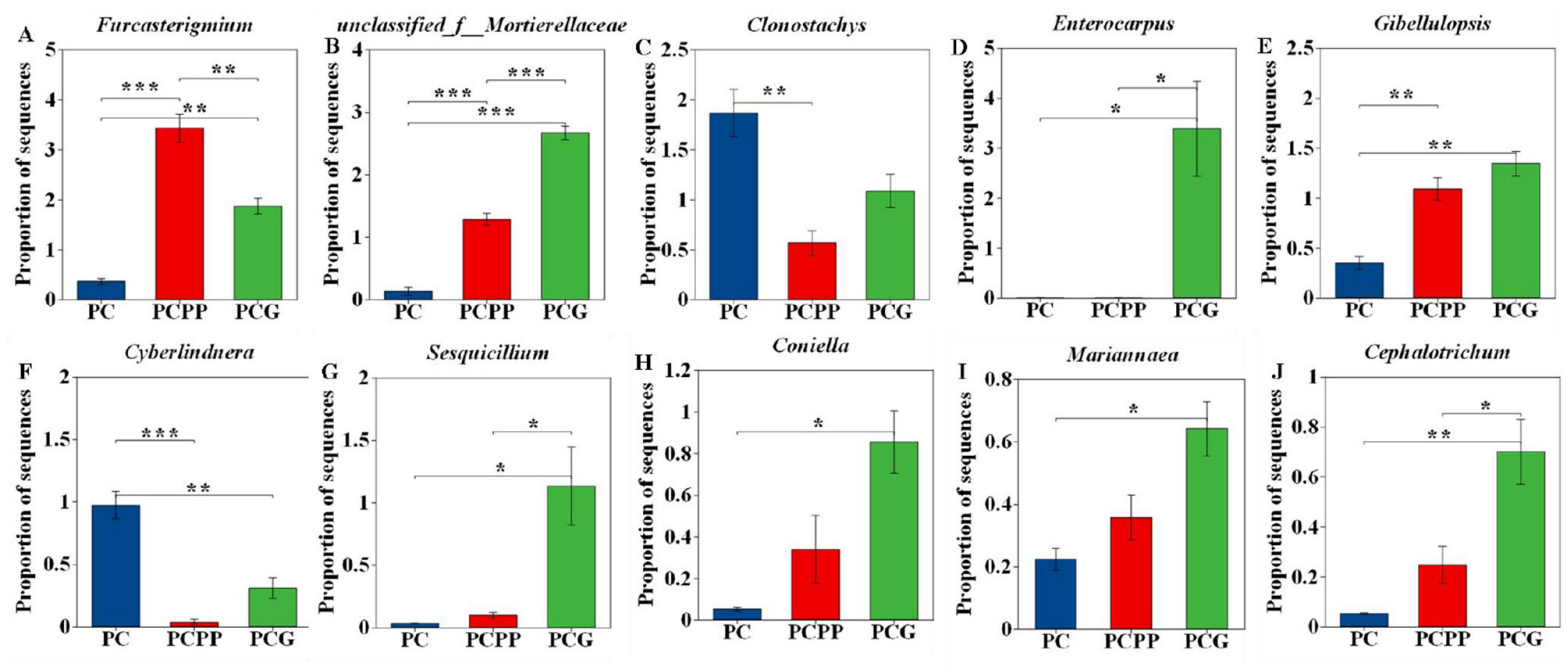
Relative abundance changes of differential fungi in the rhizosphere soil of *P. cyrtonema* under different intercropping patterns. **(A-J)** Genus level. PC, Monoculture of *P. cyrtonema*; PCPP, Intercropping of *P. cyrtonema* and *P. polyphylla*; PCG, Intercropping of *P. cyrtonema* and *G. lucidum*. * indicate significant differences (*P < 0.05; **P < 0.01; ***P < 0.001).

No significant differences were detected in fungal abundance at the phylum level across the various three cultivation systems. However, at the genus level, 63 fungal genera demonstrated statistically significant variations in relative abundance among the treatments (*P* < 0.05). The top 10 most variable genera were subjected to further analysis (Figure 5). Notably, *Furcasterigmium, unclassified_f__Mortierellaceae*, and *Gibellulopsis* showed substantially higher relative abundances in PCPP (3.43%, 1.28%, and 1.09%, respectively) and PCG treatments (1.87%, 2.67%, and 1.34%) compared to PC (0.36%, 0.13%, and 0.35%) (Figures 5A, B, E). *Clonostachys* and *Cyberlindnera* displayed higher relative abundances in PC than in PCPP, while *Cyberlindnera* showed substantially higher relative abundances in PC than in PCG (Figures 5C, F). Conversely, within the PCG treatment, *Enterocarpus, Sesquicillium*, and *Cephalotrichum* were significantly more abundant than in PC and PCPP, with relative abundance increases of 100%, 3334.65%, and 1245.52% relative to PC, respectively (Figures 5D, G, J). The relative abundances of *Coniella* and *Mariannaea* were significantly higher in PCG than in PC, with increases of 1564.07% and 186.77%, respectively. (Figures 5H, I).

**Figure 6:**
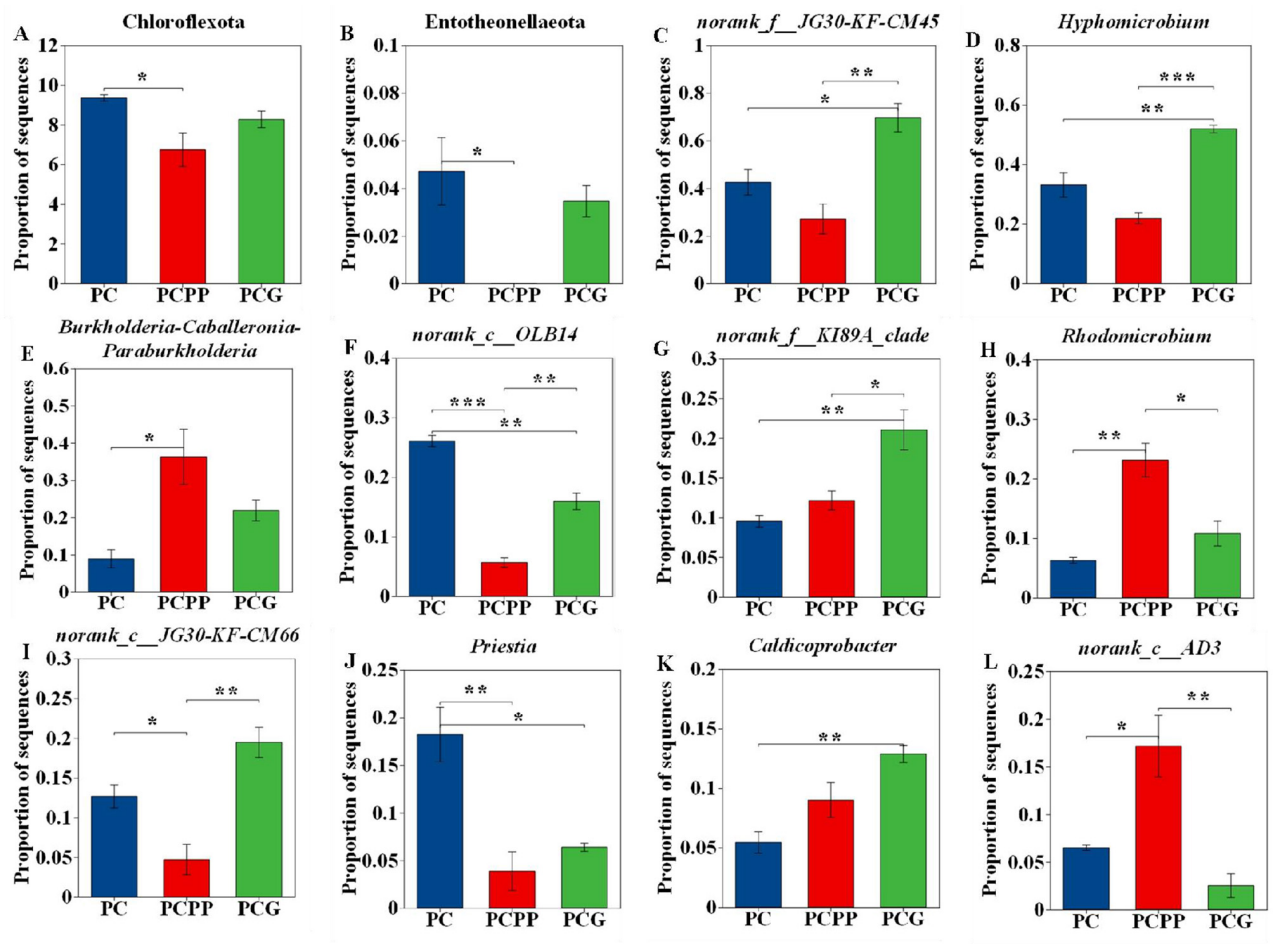
Changes in relative abundance of differential bacteria in rhizosphere soil of *P. cyrtonema* under different intercropping systems. **(A,B)** Phylum level; **(C-L)** Genus level. PC, Monoculture of *P. cyrtonema*; PCPP, Intercropping of *P. cyrtonema* and *P. polyphylla*; PCG, Intercropping of *P. cyrtonema* and *G. lucidum*. *indicate significant differences (*P < 0.05; **P < 0.01; ***P < 0.001).

At the phylum level within Bacteria, the relative abundances of Chloroflexota and Entotheonellaeota were significantly higher in the PC treatment than in the PCPP treatment (Figures 6A, B). Genus-level analysis identified significant differences in the relative abundance of 58 bacterial genera (*P* < 0.05). For further investigation, the top 10 genera with the highest relative abundances were selected (Figures 6C-L). Within the PCG treatment, *norank_f__JG30-KF-CM45, Hyphomicrobium*, and *norank_f__KI89A_clade* were significantly more abundant than in both PC and PCPP treatments, whereas *Caldicoprobacter* exhibited increased abundance relative to PC. In contrast, the PCPP treatment showed enrichment of *Burkholderia-Caballeronia-Paraburkholderia, Rhodomicrobium*, and *norank_c_AD3* compared to PC and PCG. The genus *norank_c_OLB14* demonstrated the highest abundance in the PC treatment, followed by PCG and PCPP. Additionally, *norank_c_JG30-KF-CM66* and *Priestia* were significantly more abundant in PC than in PCPP, with *Priestia* exhibiting greater abundance than in PCG.

### 3.7 Relationships among soil microbial composition and agronomic traits, bioactive components of *Polygonatum cyrtonema*, and soil physicochemical properties

**Figure 7:**
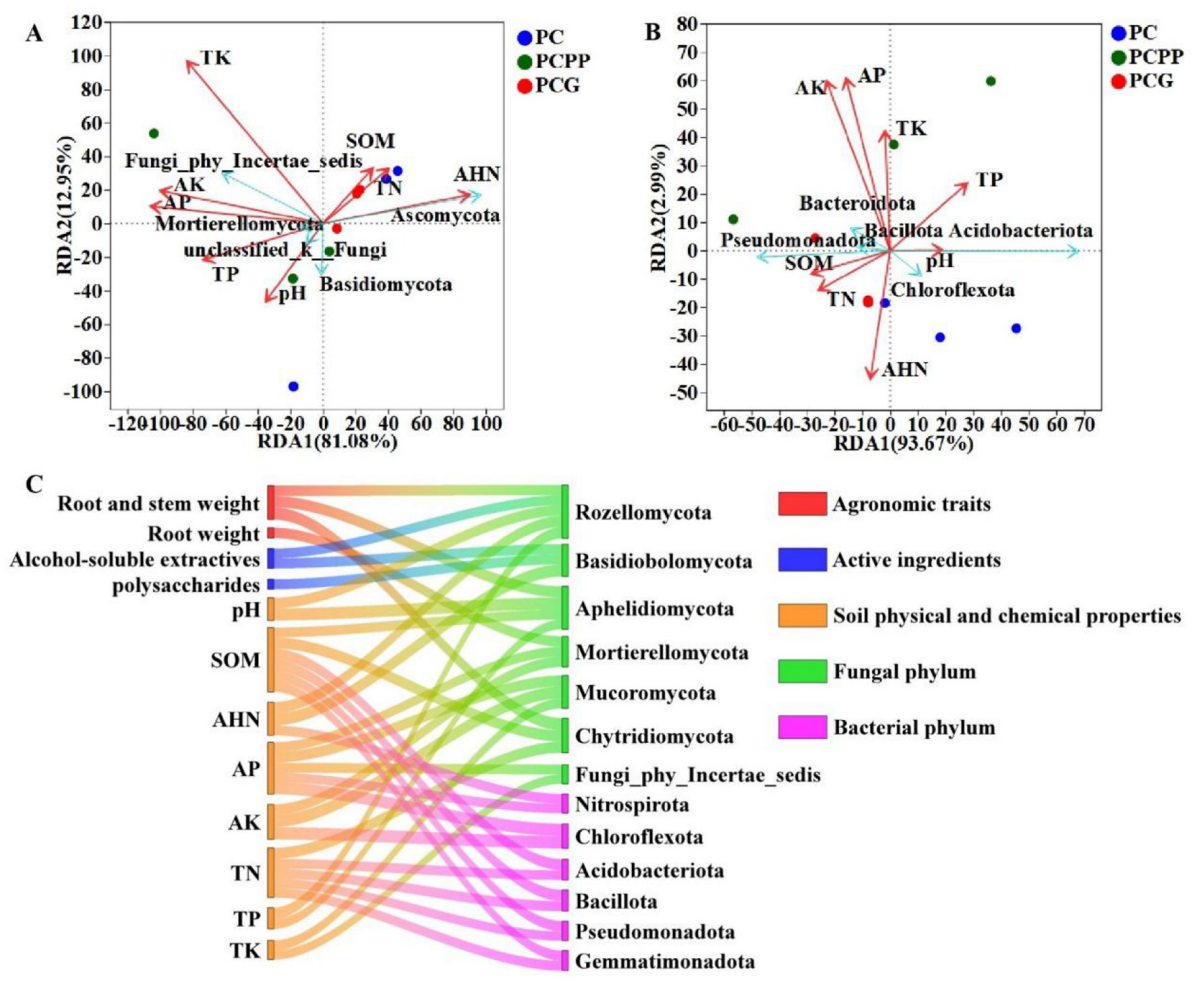
Analysis of the relationship between soil microbial community composition and the agronomic traits, active ingredients, and soil physicochemical properties. **(A)** Phylum-level RDA analysis of fungi; **(B)** Phylum-level RDA analysis of bacteria; **(C)** Sankey diagram among rhizosphere soil microenvironment, agronomic traits and components of *P. cyrtonema*. PC, Monoculture of *P. cyrtonema*; PCPP, Intercropping of *P. cyrtonema* and *P. polyphylla*; PCG, Intercropping of *P. cyrtonema* and *G. lucidum*.

Redundancy analysis (RDA) examining the ten most prevalent fungal phyla in relation to soil physicochemical parameters demonstrated that the first two axes at the phylum level accounted for 94.03% of the total variance (Figure 7A). Notably, the relative abundances of Ascomycota, Basidiomycota, and Fungi_phy_Incertae_sedis showed significant associations with specific soil properties (Figure 7A). Among these, the vector corresponding to total potassium content (TK) was the longest and exhibited the highest coefficient of determination (r^2^ = 0.6368), indicating that TK is a key factor influencing the relative abundance of dominant fungal phyla (Figure 7A).

At the bacterial phylum level, the first two RDA axes explained 96.66% of the observed variance. Soil physicochemical characteristics were correlated with the relative abundances of Pseudomonadota, Acidobacteriota, and Chloroflexota (Figure 7B). The vectors representing available potassium (AK) and available phosphorus (AP) were the longest,with the highest r^2^ values (0.8767 and 0.8445, respectively), suggesting that AK and AP are principal soil variables affecting the relative abundance of dominant bacterial phyla (Figure 7B).

To further investigate the influence of intercropping patterns on the agronomic traits and quality of *P. cyrtonema* via modulation of the soil microenvironment, a Sankey diagram was constructed to visually depict the correlations between these traits and the metabolic pathways (Figure 7C). The thickness of the connecting lines corresponds to the strength of the correlations, with thicker lines indicating stronger associations. A significant positive correlation was observed between TK and fungi classified as Fungi_phy_Incertae_sedis (*R* = 0.6667, *P* < 0.05) (Figure 7C, Supplementary Figure S2A). Similarly, Both AK (*R* = 0.8767, *P* < 0.01) and AP (*R* = −0.8721, *P* < 0.01) exhibited significant negative correlations with Chloroflexota (Figure 7C, Supplementary Figure S2B), indicating that soil concentrations of AK and AP substantially influence bacterial community structure.

Regarding agronomic traits, rhizome weight was significantly negatively correlated with Mortierellomycota (*R* = −0.7448, *P* < 0.05) (Figure 7C, Supplementary Figure S2C). Both rhizome fresh weight (*R* = 0.750, *P* < 0.05) and ethanol-soluble extract content (*R* = 0.695, *P* < 0.05) showed significant positive correlations with Rozellomycota (Figure 7C, Supplementary Figure S2C). Conversely, rhizome fresh weight was strongly negatively correlated with Chytridiomycota (*R* = −0.8833, *P* < 0.01) and a significant positive correlation with Aphelidiomycota (*R* = 0.800, *P* < 0.01) (Figure 7C, Supplementary 2C). Additionally, ethanol-soluble extract content (*R* = −0.7289, *P* < 0.05) and polysaccharide content (*R* = −0.7167, *P* < 0.05) were significantly negatively correlated with Basidiobolomycota (Figure 7C, Supplementary Figure S2C). In contrast, no significant correlations were detected between rhizome length, rhizome fresh weight, or the active components of *P. cyrtonema* and the relative abundance of the dominant soil bacterial phyla (Figure 7C, Supplementary Figure S2D).

### 3.8 Effects of intercropping on the co-occurrence network of rhizosphere soil microbial communities of *P. cyrtonema*

**Figure 8:**
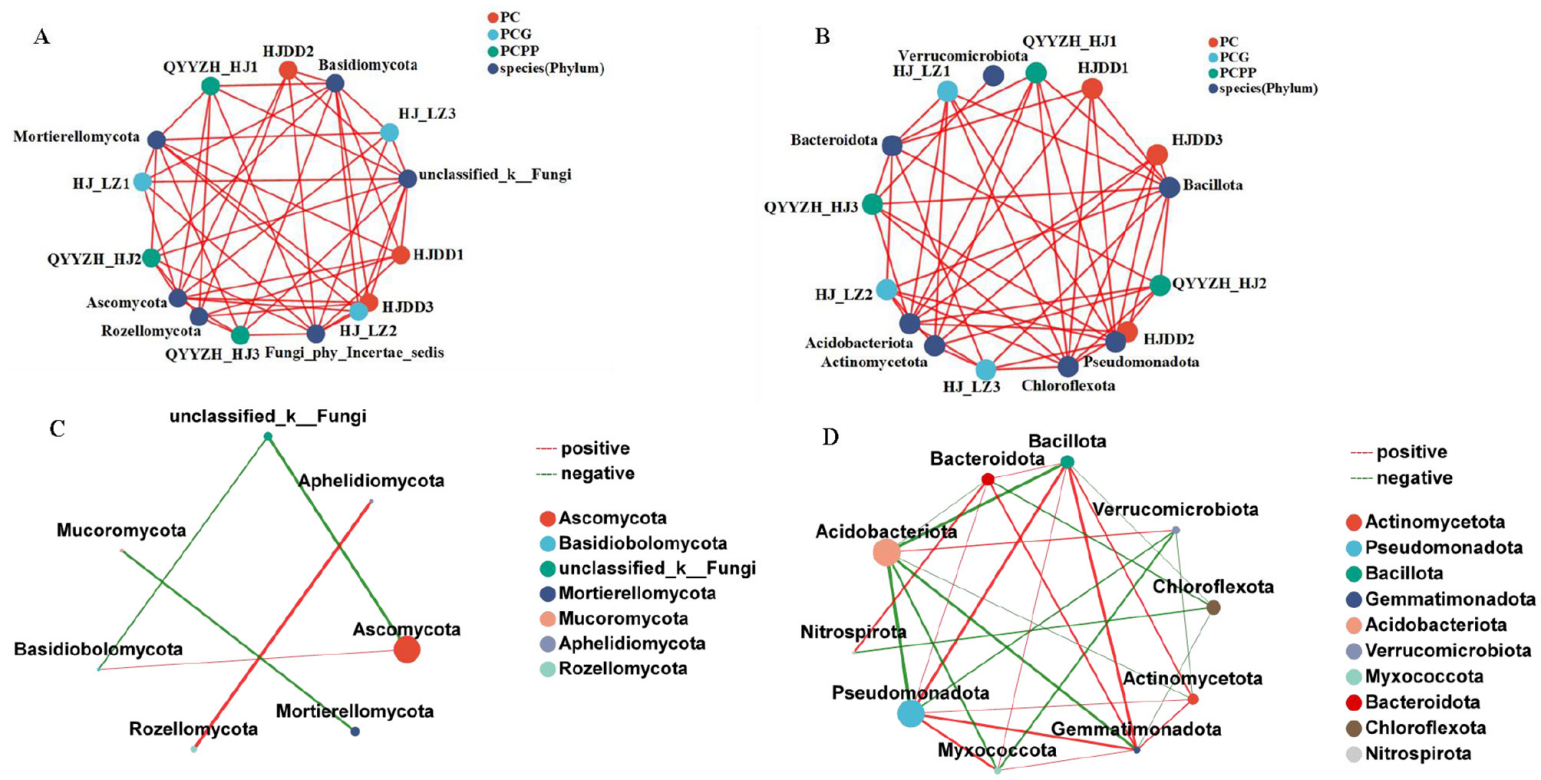
Effects of intercropping on the co-occurrence network of rhizosphere soil microbial communities of *P. cyrtonema*. **(A,C)** Fungal phylum level; **(B,D)** Bacterial phylum level.

To investigate the co-occurrence patterns within the rhizosphere microbial communities of *P. cyrtonema*, co-occurrence networks at the phylum level were constructed by selecting bacterial and fungal taxa with species abundance weight values equal to or exceeding 50. The number of edges, average degree, and average clustering coefficient of bacteria were all higher than those of fungi (Figures 8C, D; Table 2), indicating that the network structure of bacteria is more complex than that of fungi. No significant differences were observed in the network nodes representing fungal (Figure 8A) and bacterial phyla (Figure 8B) across the different treatments. Nevertheless, centrality metrics-including Degree Centrality, Closeness Centrality, and Betweenness Centrality-were notably higher for nodes corresponding to dominant phyla such as Ascomycota, Basidiomycota, unclassified_k__Fungi, Acidobacteriota, Chloroflexota, Pseudomonadota, and Bacillota compared to other taxa (Figures 8C, D). This finding suggests that these predominant phylum-level taxa occupy pivotal positions within the microbial community interaction networks.

**Table 2:** Summary of key parameters of networks.

**Topological properties**	**Fungal**	**Bacterial**
Number of nodes	5	10
Number of edges	7	27
Average degree	1.43	5.40
Average clustering coeffcient	−0.17	0.03

## 4 Discussion

### 4.1 Impacts of intercropping systems on the quality of *P. cyrtonema*

This study assessed the influence of intercropping *P. cyrtonema* with *P. polyphylla* and *G. lucidum* on both yield and quality attributes. Although intercropping did not produce statistically significant changes in yield, the PCPP system markedly improved quality parameters of *P. cyrtonema*. Specifically, concentrations of ethanol-soluble extractives and polysaccharides were elevated in the PCPP system relative to the monoculture (PC). These results corroborate recent findings by Chen F. et al. (2023), who reported enhanced polysaccharide accumulation under forest intercropping conditions. The observed improvements are likely attributable to diminished competition among rhizomes and beneficial metabolic interactions between *P. cyrtonema* and *P. polyphylla* within the PCPP system, which may indirectly stimulate the biosynthesis of bioactive compounds (Guo et al., 2020). Moreover, ethanol-soluble extract content increased to 80.30% under the PCPP system, potentially due to root exudates from *P. polyphylla* facilitating mineral uptake, thereby enhancing ethanol-soluble extract content (Ma et al., 2022; Zheng et al., 2020). Collectively, these findings indicate that the PCPP system constitutes an effective strategy for enhancing the quality of *P. cyrtonema*.

### 4.2 Impacts of intercropping systems on soil physicochemical properties

Extensive research has demonstrated the beneficial effects of intercropping on soil nutrient status (Zhou et al., 2023; Wang et al., 2022). In the present study, both intercropping systems (PCPP and PCG) significantly increased SOM, AHN, AP, AK, and TN compared to the monoculture (PC). Although TP content was significantly lower under the PCPP system relative to PC and PCG systems, this system exhibited the most pronounced increases in AHN, AK, and TN. Conversely, the PCG system primarily enhanced AP and AK concentrations. These results align with prior studies demonstrating that intercropping *Coptis chinensis* with *Fritillaria thunbergii* or *Scrophularia ningpoensis* improves potassium, phosphorus, and alkali-hydrolyzable nitrogen availability in the rhizosphere soil (Bajiu et al., 2024).

Nutrient accumulation patterns were system-specific: the PCPP system predominantly enhanced nitrogen availability, with AHN and TN increasing by 7.19% and 7.02%, respectively, relative to PC and PCG systems. This enhancement may be attributed to nitrogenous compounds, such as amino acids, released from *P. polyphylla* rhizomes, which are subsequently mineralized by soil microorganisms, thereby elevating soil nitrogen content (Paynel et al., 2001). Intercropping also induces adaptive modifications in root morphology, facilitating phosphorus and other nutrient uptake. Additionally, intercropping promotes organic acid exudation and modulates soil microbial community composition, enhancing phosphorus bioavailability and resulting in decreased total soil phosphorus (Su et al., 2023). In contrast, the PCG system more effectively mobilized phosphorus and potassium, with AP and AK increasing by 23.20% and 72.76%, respectively, compared to the PC system. This effect is likely due to organic acid secretion (e.g., citric acid), by *G. lucidum* mycelia, which solubilize insoluble phosphorus and release potassium from clay minerals (Han et al., 2022; Wen et al., 2023). Importantly, both intercropping systems maintained soil pH within a slightly acidic range (6.49–6.60), consistent with the optimal pH for *P. cyrtonema* growth (5.5–6.5), thereby preventing soil acidification or alkalization (Goulding, 2016), fostering a stable soil environment conducive to plant development.

### 4.3 Impacts of intercropping on rhizosphere microbial communities

Intercropping systems significantly influence the rhizosphere microbial community of *P. cyrtonema* by modifying the microenvironment, particularly through modifications in nutrient availability and root exudate composition (Wang et al., 2024). Fungal communities exhibit greater sensitivity to these environmental changes than bacterial communities (Pascale et al., 2020). Although overall microbial diversity was not markedly affected by intercropping, fungal richness was highest in the PCG system, followed by PCPP, and lowest in PC. This pattern may be explained by additional carbon sources supplied by *G. lucidum* exudates (Wang et al., 2020). Beta diversity analyses revealed that fungal communities in the PCPP and PCG systems were similar but distinct from those in PC, indicating ecological niche complementation in intercropping systems. In contrast, bacterial communities differed significantly across all three cultivation systems.

Intercropping notably altered the relative abundance of specific microbial genera, especially within dominant fungal phyla such as Ascomycota and Basidiomycota. These phyla also exhibited higher centrality values in co-occurrence network analysis, underscoring their roles as key hubs linking soil nutrients and plant metabolism. Relative abundances of *Gibellulopsis* and *Mariannaea*, both members of the Ascomycota phylum, were significantly elevated in intercropping systems compared to monoculture system. Ascomycota fungi are recognized for decomposing recalcitrant organic compounds such as lignin and cutin, thereby playing critical roles in carbon and nitrogen cycling (Clemmensen et al., 2013). Moreover, the abundance of *unclassified_f_Mortierellaceae* (belonging to the Mortierellomycota phylum) was higher in the intercropping systems (PCPP and PCG) than in monoculture. This taxon is implicated in organic matter decomposition, phosphate solubilization, phytohormone and lipid biosynthesis, disease suppression, and soil structure improvement (Koechli et al., 2018; Brabcová et al., 2016).

Changes in bacterial community composition were closely linked to nitrogen, phosphorus, and potassium dynamics (Liang et al., 2024; Zou et al., 2022; Wu et al., 2021). At the genus level, the PCG system significantly increased the relative abundances of *norank_f_JG30-KF-CM45* and *Hyphomicrobium* by 63.77% and 56.96%, respectively, compared to PC. These genera are associated with denitrification (Bhatia et al., 2013) and phosphate solubilization (Shen et al., 2023). In contrast, the PCPP system exhibited higher abundance of *Burkholderia-Caballeronia-Paraburkholderia* relative to PC. This group includes several recognized nitrogen-fixing species (Ding et al., 2024). This observation aligns with the increased soil nitrogen observed in the PCPP system, supporting the notion that functional microbial taxa correspond to nutrient availability (Jiao et al., 2021).

Redundancy analysis revealed that fungal community structure was primarily influenced by total potassium (TK), which is essential for microbial membrane integrity and enzymatic activity (Yu et al., 2023). In contrast, bacterial community structure was predominantly regulated by available potassium (AK) and available phosphorus (AP), which are readily utilized by microbes and plants, thereby selecting for taxa adapted to specific nutrient conditions. These findings indicat that intercropping systems modulate key soil nutrients (TK, AK, and AP) to shape rhizosphere microbial composition, thereby establishing a favorable microecological environment that promotes the growth and quality of *P. cyrtonema*.

### 4.4 Impacts of rhizosphere soil microenvironment on the quality of *P. cyrtonema*

Variations in the rhizosphere soil microenvironment significantly affect the quality of *P. cyrtonema* by influencing nutrient transformation and metabolic regulation, with fungal taxa exerting a more direct influence. Correlation analyses demonstrated that the abundance of Rozellomycota was positively associated with both rhizome fresh weight and ethanol-soluble extract content (Figures 7C, Supplementary Figure S2C), suggesting a role in enhancing nutrient uptake and promoting biosynthesis of bioactive compounds (Mei et al., 2023; Zhang et al., 2025). In contrast, increased abundance of Mortierellomycota was negatively correlated with rhizome biomass, implying potential nutrient competition that may impede quality development (Peng et al., 2024). Although relationships between bacterial taxa and quality parameters were not significant, the proliferation of functional groups such as Gemmatimonadota and Bacteroidota may improve nutrient availability (e.g., AP and AK), thereby indirectly supporting quality enhancement. This observation aligns with the conceptual framework proposed by Banerjee et al. (2018), which posits that microbial communities regulate plant productivity through modulation of soil nutrient dynamics.

Specifically, under the PCPP mode, soil AHN and TN contents increased (Figures 3C, F), concomitant with enrichment of the nitrogen-fixing genus *Burkholderia-Caballeronia-Paraburkholderia* (Figure 6E). This genus can decompose insoluble organic nitrogen, augment soil nitrogen availability, facilitate root absorption, and directly promote rhizome polysaccharide synthesis (Ding et al., 2024; Conners et al., 2024; Deng et al., 2018). Under the PCG mode, differential fungi such as *unclassified_f__Mortierellaceae* secrete organic acids, which activate soil mineral elements and increase soil AK and AP (Figures 3D, E; Sang et al., 2022; Friedrich et al., 1991). Phosphorus and potassium are critical for photosynthesis and facilitate transport of photosynthates to rhizomes, thereby enhancing ethanol-soluble extract content (Ma et al., 2024; Peng et al., 2018). Therefore, the combined effects of altered soil nutrients and rhizosphere microbial communities collectively regulate the accumulation of active components in *P. cyrtonema* rhizomes, resulting in variations in quality.

## 5 Conclusions

The findings of this study demonstrate that the PCPP intercropping system promotes the accumulation of active constituents in *P. cyrtonema* and enhances soil nutrient availability. This system also modifies the structure of rhizosphere microbial communities, which are closely associated with soil nutrient status. Specifically, soil total potassium (TK) was identified as a key factor shaping fungal community composition, while available potassium (AK) and available phosphorus (AP) predominantly influenced bacterial community structure. These interactions collectively established a favorable microecological environment conducive to medicinal plant growth. However, as this study was conducted in a sandy soil context, the generalizability of the results may be constrained by soil and climate conditions. Consequently, future research should undertake multi-site experiments across different locations to further develop a comprehensive management plan for the high-quality and efficient cultivation of *P. cyrtonema*.

## Data Availability

The datasets presented in this study can be found in online repositories. The names of the repository/repositories and accession number(s) can be found in the article/Supplementary material.
