# Supplementary material for: Intercropping with Paris polyphylla and Ganoderma lucidum: regulatory effects on the rhizosphere microbial community and the quality of Polygonatum cyrtonema
Source: Front Microbiol. 2025 Dec 17;16:1711104. doi: 10.3389/fmicb.2025.1711104 (PMC12756931; doi:10.3389/fmicb.2025.1711104)
Supplement: Supplementary file 1 [file Data_Sheet_1.docx]

**Table S1 Alpha diversity indices of microbial communities in the rhizosphere soil of *P. cyrtonema* across various different intercropping systems**

| Fungal species diversity | | | | | | |  | Bacterial species diversity | | | | | |
| --- | --- | --- | --- | --- | --- | --- | --- | --- | --- | --- | --- | --- | --- |
| Sample | Ace index | Chao index | Shannon index | Simpson index | Coverage(%) | Sobs index |  | Ace index | Chao index | Shannon index | Simpson index | Coverage(%) | Sobs index |
| PC | 745.16±86.97a | 744.49±86.91a | 4.50±0.26a | 0.03±0.01a | 99.98 | 742.33±85.16a |  | 1747.83±132.679a | 1736.47±122.02a | 6.84±0.02a | 0.00±0.00a | 99.82 | 1725.33±112.84a |
| PCPP | 758.17±31.12a | 756.95±30.86a | 4.41±0.22a | 0.05±0.01a | 99.98 | 755.67±30.89a |  | 1631.89±74.62a | 1621.72±70.77a | 6.77±0.03a | 0.00±0.00a | 99.87 | 1617.00±68.02a |
| PCG | 809.49±47.79a | 807.99±47.48a | 4.47±0.19a | 0.05±0.01a | 99.98 | 806.67±48.00a |  | 1676.40±137.16a | 1665.92±126.84a | 6.81±0.03a | 0.00±0.00a | 99.86 | 1659.00±116.11a |

^1^ PC: Monoculture of *P. cyrtonema*; PCPP: Intercropping of *P. cyrtonema* and *P. polyphylla*; PCG: Intercropping of *P. cyrtonema* and *G. lucidum*.

^2^ Different lowercase letters within the same column indicate statistically significant differences (*P* < 0.05).


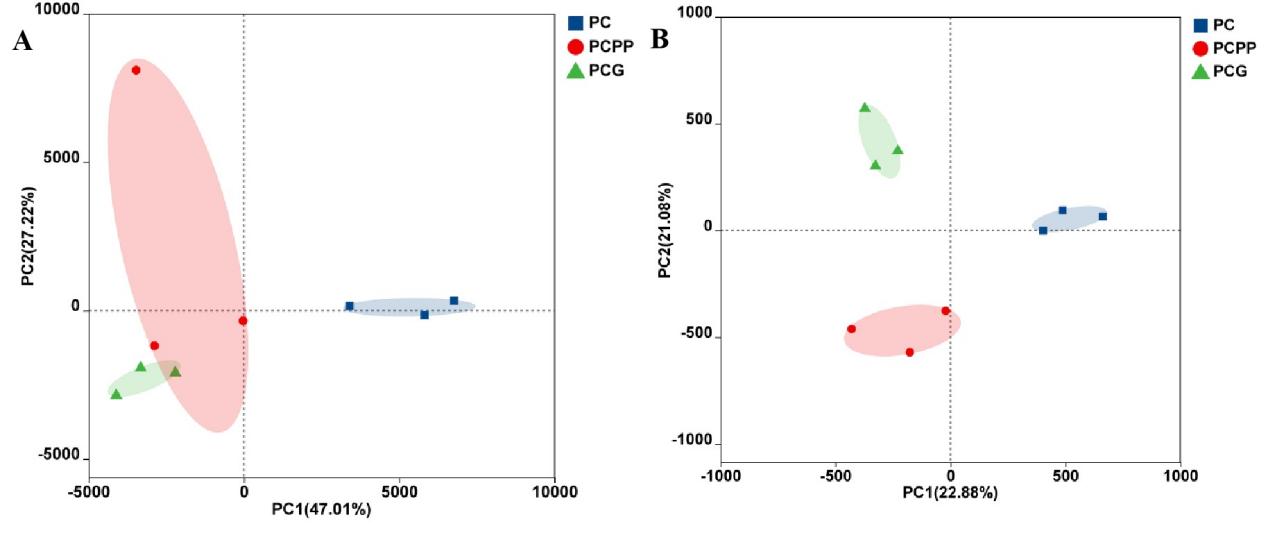


**Figure S1.** The influence of various intercropping systems on microbial species diversity. **A**: Fungal species diversity; **B**: Bacterial species diversity. PC: Monoculture of *P. cyrtonema*; PCPP: Intercropping of *P. cyrtonema* and *P. polyphylla*; PCG: Intercropping of *P. cyrtonema* and *G. lucidum*.


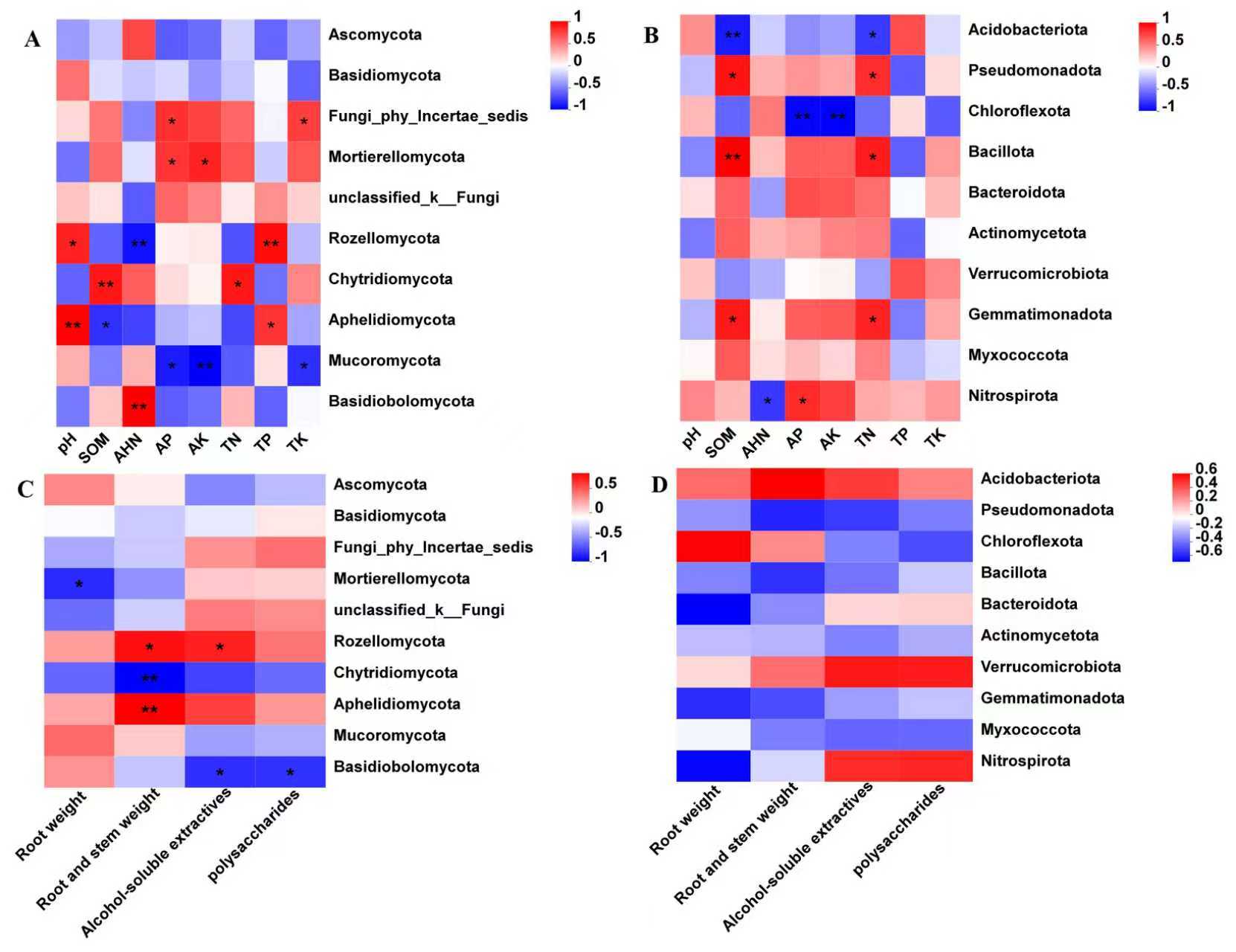


**Figure S2** The relationship between soil physicochemical characteristics and the dominant soil fungal phylum composition (**A**) and the dominant soil bacterial phylum (**B**); Correlation between the active components of *P. cyrtonema* and the dominant soil fungal phyla (**C**) and the dominant soil bacterial phyla (**D**). * indicate significant differences (*: *P* < 0.05; **: *P* < 0.01).
